# Second-line tests in the differential diagnosis of neoplastic and non-neoplastic hypercortisolism: a systematic review and meta-analysis

**DOI:** 10.1007/s40618-023-02099-z

**Published:** 2023-04-20

**Authors:** A. Mondin, M. Barbot, G. Voltan, I. Tizianel, C. K. Vedolin, P. Mazzeo, M. Lazzara, M. Boscaro, C. Scaroni, F. Ceccato

**Affiliations:** 1 Endocrinology Unit, Department of Medicine DIMED, University of Padova, Via Ospedale Civile, 105, 35128 Padua, Italy; 2 Endocrine Disease Unit, University-Hospital of Padova, Padua, Italy; 3 Department of Neuroscience DNS, University of Padova, Padua, Italy

**Keywords:** Pseudo-Cuhing, Non-neoplastic hypercortisolism, ACTH-dependent Cushing’s syndrome, Cushing’s disease, CRH test, Desmopressin test, Dexamethasone

## Abstract

**Purpose:**

The clinical and hormonal overlap between neoplastic (CS) and non-neoplastic (NNH/pCS) hypercortisolism is a challenge. Various dynamic tests have been proposed to allow an early discrimination between these conditions, but to date there is no agreement on which of them should be used.

**Aim:**

To provide an overview of the available tests and to obtain a quantitative synthesis of their diagnostic performance in discriminating NNH/pCS from CS.

**Methods:**

The included articles, published between 1990 and 2022, applied one or more second line tests to differentiate NNH/pCS from CS patients. For the NNH/pCS group, we admitted the inclusion of patients presenting clinical features and/or biochemical findings suggestive of hypercortisolism despite apparent lack of a pCS-related condition.

**Results:**

The electronic search identified 339 articles. After references analysis and study selection, we identified 9 studies on combined dexamethasone-corticotropin releasing hormone (Dex-CRH) test, 4 on Desmopressin test and 3 on CRH test; no study on Dex-Desmopressin met the inclusion criteria. Dex-CRH test provided the highest sensitivity (97%, 95 CI% [88%; 99%]). CRH tests showed excellent specificity (99%, 95% CI [0%; 100%]), with low sensitivity. Although metaregression analysis based on diagnostic odds ratio failed to provide a gold standard, CRH test (64.77, 95% CI [0.15; 27,174.73]) seemed to lack in performance compared to the others (Dex-CRH 138.83, 95% CI [49.38; 390.32] and Desmopressin 110.44, 95% CI [32.13; 379.63]).

**Discussion:**

Both Dex-CRH and Desmopressin tests can be valid tools in helping discrimination between NNH/pCS and CS. Further studies are needed on this topic, possibly focusing on mild Cushing’s Disease and well-characterized NNH/pCS patients.

**Systematic review registration:**

https://www.crd.york.ac.uk/prospero/display_record.php?ID=CRD42022359774, identifier CRD42022359774.

## Introduction

Many conditions (e.g., psychiatric disorders, alcoholism, obesity, eating disorders or polycystic ovary syndrome) can cause a functional activation of the hypothalamic–pituitary–adrenal (HPA) axis. This condition, formerly known as pseudo-Cushing (pCS) state, is nowadays better addressed as non-neoplastic hypercortisolism (NNH) [1] and its differentiation from the neoplastic form (i.e., Cushing syndrome, CS) represents a clinical and biochemical diagnostic challenge for physicians.

In CS, some clinical features (such as bruisability, facial plethora, proximal myopathy and large purple cutaneous striae) are highly specific, but they lack sensitivity; vice versa other features are very common also in the general population and in NNH/pCS patients [2].

Endocrine society guidelines [3] recommended using either urine free cortisol (UFC), late-night salivary cortisol (LNSC) or low-dose dexamethasone suppression test (DST) to assess biochemical endogenous hypercortisolism. Both 1 mg overnight and longer (2 mg/days for 48 h) dexamethasone suppression could be considered, although the more recent consensus on Cushing’s Disease (CD) [4] only reported the 1 mg DST. The combination of multiple tests should be used to confirm CS diagnosis, and their application should consider both their strengths (availability, ease collection, high diagnostic accuracy) and pitfalls (concomitant medications, altered sleep–wake rhythm, intra-personal variability, laboratory assays) [5]. In a recent metanalysis from Galm et al. [6], all of the included diagnostic tests for CS proved highly sensitive and specific; still DST seemingly provided the highest sensitivity and UFC the lowest. The above-mentioned results were obtained evaluating subjects with clinical suspicion of hypercortisolism. Nevertheless, patients with non-neoplastic HPA axis hyperactivation may present with the biochemical evidence of a mild hypercortisolism at first-line tests, largely indistinguishable from that of a CS patient.

As clinical and biochemical profiles frequently overlap in NNH/pCS and CS, various dynamic tests had been proposed to guide this differential diagnosis. A combined dexamethasone-corticotropin releasing hormone (Dex-CRH) test was first proposed by Yanovski et al. [7] to discriminate NNH/pCS from CS patients, based on the hypothesis that only the latter could maintain cortisol response to CRH stimulation even after prior dexamethasone suppression. CRH test alone is widely used to diagnose hormone excess source in ACTH-dependent CS [4], but it was also studied to discern functional and neoplastic hypercortisolism. More recently desmopressin test emerged as a potential tool to discriminate NNH/pCS and CS subjects, based on the different pattern of vasopressin receptors expression in pituitary corticotroph adenomas compared to the normal pituitary [8]. The use of desmopressin stimulus might be chosen as less expensive alternative to CRH after dexamethasone suppression (i.e., Dex-desmopressin instead of Dex-CRH test) [9]. To date there is no agreement on which test should be used in this complicated setting.

The aim of this systematic review and metanalysis is to provide an overview of the available tests and to obtain a quantitative synthesis of their diagnostic performance in order to differentiate patients with NNH/pCS from those with CS.

## Materials and methods

**Fig. 1:**
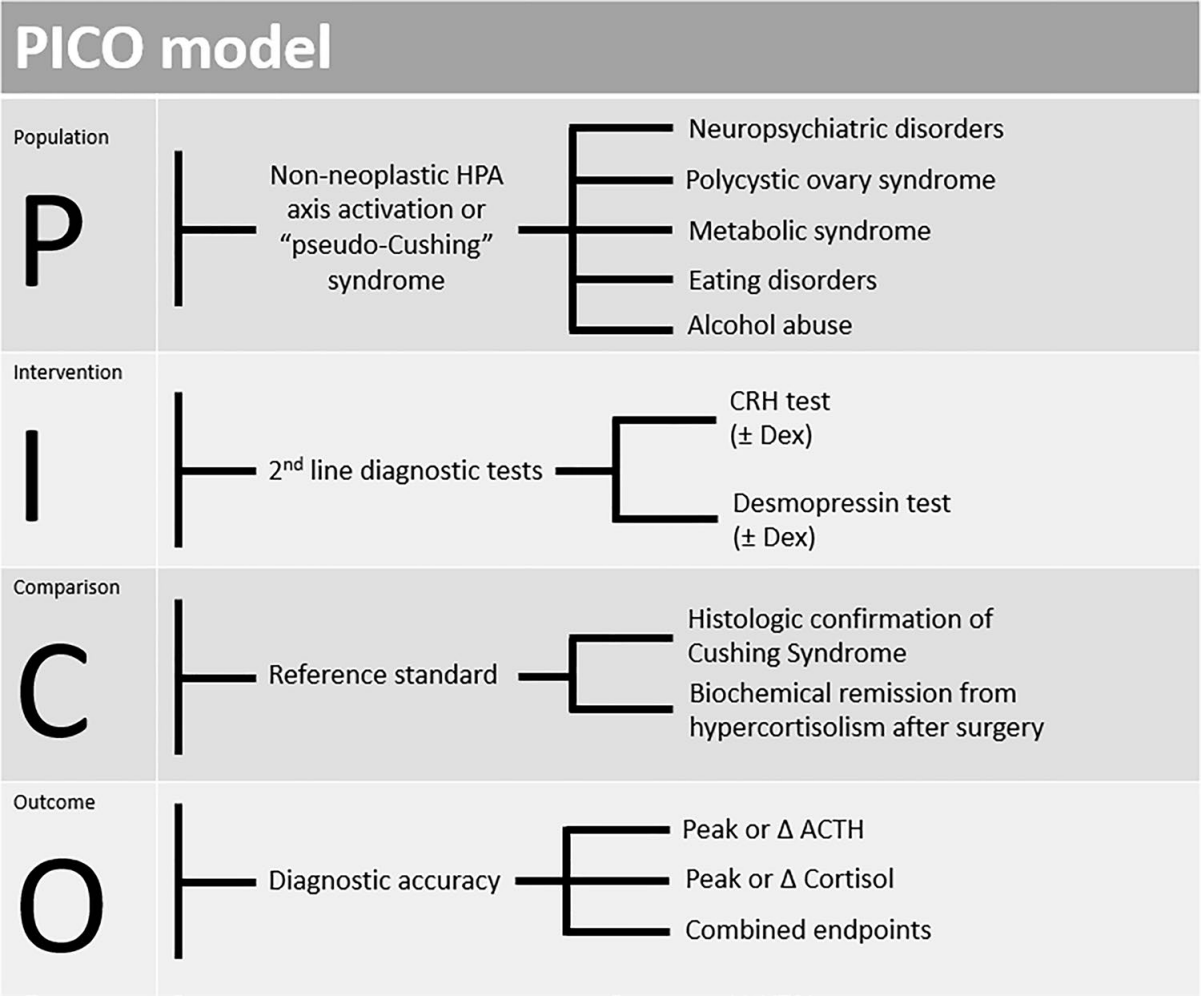
PICO (Population–Intervention–Comparison–Outcome) model design for our study. *HPA* hypothalamic pituitary adrenal, *CRH* corticotropin releasing hormone, *Dex* dexamethasone

We used the Population-Intervention-Comparison-Outcome (PICO) model to formulate the research question for the systematic review [10], as summarised in Fig. 1. The systematic review and meta-analysis were conducted and are reported according to the Preferred Reporting Items for Systematic Reviews and Meta-Analysis of Diagnostic Test Accuracy Studies (PRISMA-DTA) statement [11]. We registered the protocol on the International Prospective Register of Systematic Reviews database (PROSPERO, https://www.crd.york.ac.uk/PROSPERO, number CRD42022359774).

### Search strategy

An extensive search in Ovid (including Cochrane, Medline and Embase databases) was performed for the research question by two of the authors (A.M. and F.C.) independently, discrepancies were resolved by discussion. Literature search was performed from January 1990 up to May 2022, no language restriction was applied. Research included the following key words: (pseudocushing$.mp. OR pseudo-cushing$.mp. OR non-neoplastic hypercortisolism$.mp. OR non neoplastic hypercortisolism$.mp. OR Neuropsychiatric disorder$.mp. OR Polycystic ovary syndrome$.mp. OR Metabolic syndrome$.mp OR Eating disorder$.mp. OR Alcohol abuse$.mp.) AND (CRH test$.mp. OR corticotropin releasing hormone test$.mp. OR Dex-CRH test$.mp. OR dexamethasone-suppressed corticotropin-releasing hormone stimulation test$.mp. OR combined dexamethasone-corticotropin releasing hormone test$.mp. OR ddavp test$.mp. OR desmopressin test$.mp. OR Dex-Desmopressin test$.mp. OR dexamethasone-suppressed desmopressin stimulation test$.mp).

Specific inclusion and exclusion criteria were specified in advance and protocol-defined in order to avoid methodological bias for post-hoc analysis. The search was designed to select all types of studies (retrospective, observational, controlled randomized and non-randomized trials) evaluating the accuracy of one or more second line tests in distinguishing patients with NNH/pCS (control group) from those presenting CS (case group). Regarding the control group, we admitted the inclusion, along with adequately characterized NNH/pCS patients, of subjects presenting clinical features and/or biochemical findings suggestive of hypercortisolism despite the apparent lack of a pCS-related condition (i.e., “CS-excluded” patients). On the contrary, studies including subjects without clinical features and/or biochemical findings suggestive of hypercortisolism (i.e., “control” patients) were excluded. The considered dynamic tests included the CRH and the Desmopressin stimulation test, with or without previous Dex suppression.

Search terms were linked to Medical Subject Headings and when possible. Keywords and free words were used simultaneously.

Additional articles were identified with manual searches and included through a review of other meta-analyses, review articles and relevant references.

Consolidation of studies was performed with Mendeley Desktop 1.19.8.

### Study selection

We included all the original research studies applying one or more second line dynamic tests (i.e. CRH test, Dex-CRH test, Desmopressin test, Dex-Desmopressin test) to differentiate NNH/pCS from CS patients. Both cut-offs achieving best performance (ROC-based) and predefined (based on previous reports on different cohorts) were considered for each study. Inclusion of “CS-excluded” subjects in the NNH/pCS group was admitted, while control subjects were excluded (see above). Reviewers were not blinded to the authors or journals when screening articles.

### Data extraction and quality assessment

Two authors (A.M. and F.C.) read the included papers and extracted independently predefined data, any disagreements were resolved by discussion. If data were not clear from the original manuscript, the authors of the primary study were contacted in attempt to clarify their data.

Contents of data extraction in the selected paper included: name of the first author, year of publication, study design, number of patients for each category [i.e., NNH/pCS, CS-excluded, adrenal CS (ACS), CD and ectopic ACTH secreting tumor (EAS)], diagnostic test accuracy data as true positives (TP: patients with CS presenting response to the tests), true negatives (TN: patients without CS and with lack of response to the tests), false negatives (FN: patients with CS and lack of response to the tests), false positives (FP: patients without CS, but presenting response to the tests) and their derived indexes. For studies using Dex suppression prior to a stimulation, Dex schedule was reported. For stimulation tests, type (ovine or human) and dosage of CRH and dosage of Desmopressin were reported.

The Quality Assessment of Diagnostic Accuracy Studies 2 (QUADAS-2) tool was used to assess the quality of included studies [12]. Two authors (A.M. and F.C.) independently evaluated each included study for risk of bias and concerns about applicability regarding three key domains: patient selection, index test and reference standard. The risk of bias was also assessed regarding a fourth domain, considering the flow of patients through the study and the timing of the index test and reference standard. A standardized protocol for evaluation was established prior to QUADAS-2 tool application and is available in the Supplementary Data [13]. Risk of bias or concern for applicability were deemed high, low, or presenting some concerns based on it. If researchers assessed concerns outside the eventualities covered by the protocol, the judgment could be modified accordingly.

### Data synthesis and analysis

A qualitative synthesis was performed summarizing the study design and population, the diagnostic characteristics, the assays used for discriminating analytes and the testing protocol.

In order to reduce selection bias for primary analysis, studies and thresholds were included according to the following process.

1. Among studies from the same group of research, that included presumably overlapping population, we reported that with higher likelihood of subjects presenting pCS-related conditions in the control group. If the proportion of patients with pCS-related conditions in the NNH/pCS group was equal among the studies, that with a larger population was considered.
2. If different cut-offs were available for a study, ROC-based thresholds were preferred over the predefined ones (i.e., previously specified for a specific test).
3. If a study provided two or more cut-offs of the same type, the threshold presenting better sensitivity was chosen in order to favour the correct diagnosis of a rare disease (i.e., CS).

When not provided, sensitivity, specificity, diagnostic odds ratio and their 95% confidence intervals (95% CIs) were calculated from the available data.

A random-effect model was used to estimate pooled effects. Paired forest plots for sensitivity, specificity and diagnostic odds ratio were generated to visualize between-study heterogeneity. Finally, publication bias as a potential source of heterogeneity was assessed using the Deeks’ funnel plots and Egger’s regression test (where p < 0.05 was considered statistically significant). The I^2^ test was conducted to analyse the heterogeneity between studies: I^2^ > 50% indicated significant between-study heterogeneity.

Statistical analyses were performed with R: R-4.2.0 for Windows 10 (32/64 bit) released on April 2022 and R studio desktop version 4.2.0 (2022-04-22) for Windows 10 64 bit (R Foundation for Statistical Computing, Vienna, Austria, URL https://www.R-project.org/).

## Results

### Study selection

**Fig. 2:**
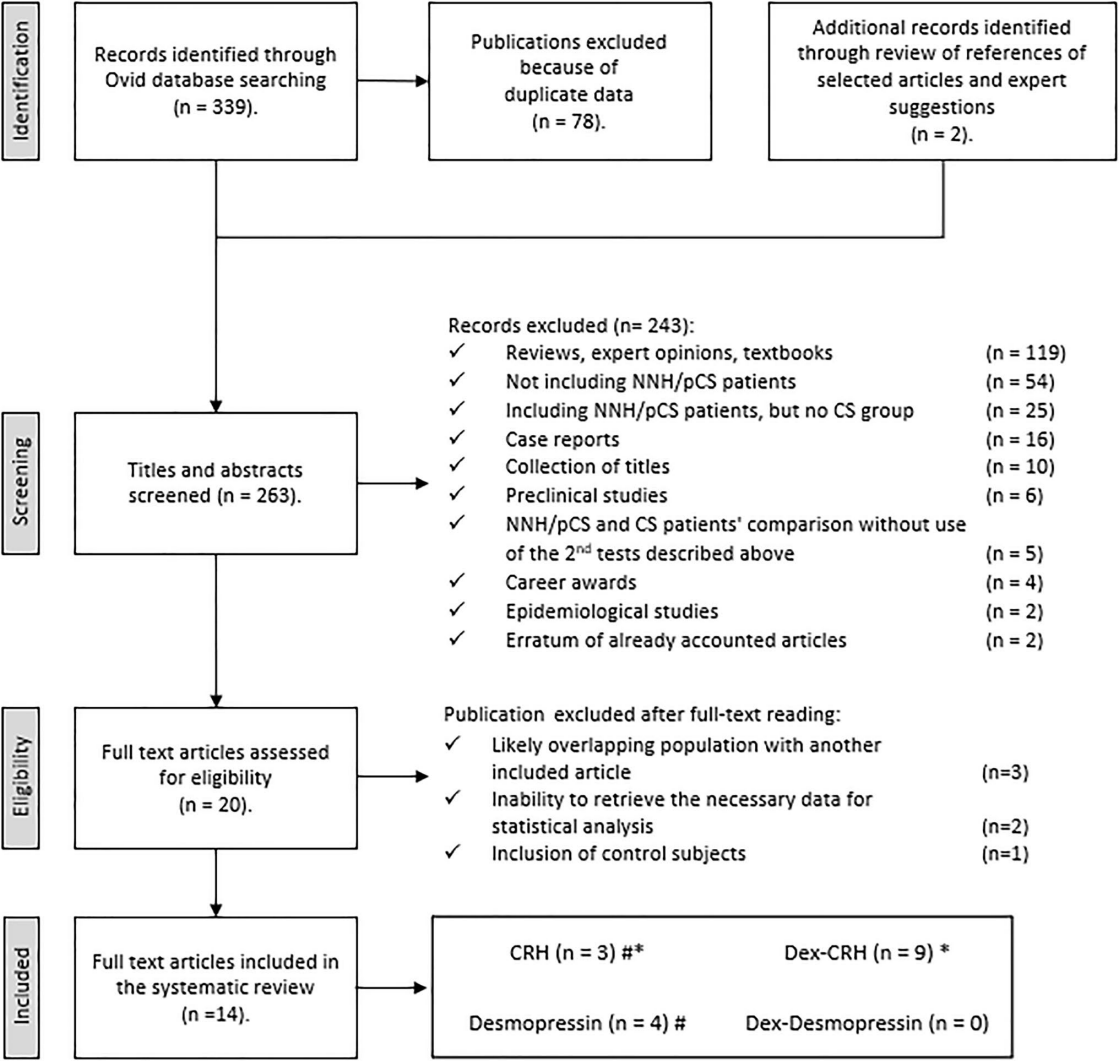
Search strategy. *NNH/pCS* non-neoplastic hypercortisolism/pseudo-Cushing, *CS* Cushing’s syndrome, *CRH* Corticotropin releasing hormone, *Dex* Dexamethasone. *One work considered both CRH and Dex-CRH test. ^#^One study considered both Desmopressin and Dex-CRH test

The study selection process is depicted in Fig. 2. Electronic search revealed 339 articles. After duplicate removal (n = 78) and considering the post-hoc inclusion of two additional papers through reference analysis of selected articles, a total of 263 of them were screened by title and abstract. After the first screening, 243 articles did not meet the eligibility criteria and were discarded for the reasons resumed in the diagram. The full text examination of the remaining studies excluded additional 6 articles: three works presented a cohort with likely overlapping patients with the population of another included study, for two studies it was not possible to retrieve the necessary data for statistical analysis (as corresponding authors did not respond to our request for elucidations on their data) and one work was excluded due to the inclusion of control subjects (i.e., no clinical or biochemical features of hypercortisolism) in the NNH/pCS group. Thus 14 studies fulfilling eligibility criteria were selected for data extraction and analysis (two studies reported more than one test) (Fig. 2). Finally, 9 studies on Dex-CRH test, 4 on Desmopressin test and 3 on CRH test were available (Tables 1, 2, 3). No study on Dex-Desmopressin met the inclusion criteria.

**Table 1:** Studies included in the meta-analysis for combined dexamethasone-corticotropin releasing hormone (Dex-CRH) test

Study	Design, setting	CS			NNH/pCS		Cut-off			Se (%)	Sp (%)	DOR	Dex	iv CRH	
		ACS	CD	EAS	pCS rc	CS ex	Type	Value	Assay					Type	Dosage
Yanovski (1993) [7]	Prospective, monocentric	2	35	2	17	2	ROC based	Cortisol at 15′: 38 nmol/L (1.4 µg/dL)	RIA	100	100	3081	0.5 mg every 6 h, 8 doses	Ovine	1 µg/kg
Martin (2006) [14]	Prospective, monocentric	4	8	0	3	21	ROC based	Cortisol at 15′: 50 nmol/L (1.8 μg/dL)	C.L. assay	100	88	154	0.5 mg every 6 h, 9 doses	Human	100 µg
Gatta (2007) [15]	Retrospective, multicentric	0	17	0	14	0	ROC based	Cortisol at 15′: 110 nmol/L (4 μg/dL) or ACTH at 15′: 3.5 pmol/L (16 pg/mL)	RIA/C.L. assay	100	86	175	0.5 mg every 6 h, 8 doses	Human/ovine*	100 µg
Erickson (2007) [16]	Retrospective, monocentric	0	21	0	30	0	ROC based	ACTH at 15′: 5.9 pmol/L (27 pg/mL)	RIA	95	97	580	0.5 mg every 6 h, 8 doses	Ovine	1 µg/kg
Pecori -Giraldi (2007) [17]	Prospective, multicentric	3	29	0	23	0	ROC based	Cortisol at 15’: 75 nmol/L (4 μg/dL)	RIA	100	87^#^	381	0.5 mg every 6 h, 8 doses	Ovine	100 µg
Batista (2008)^¥^ [18]	Retrospective, monocentric	0	11	0	11	10	ROC based	Cortisol at 15′: 88 nmol/L (3.2 μg/dL)	RIA	91	95	200	300 µg/kg every 6 h 8 doses^§^	Ovine	1 µg/kg
Reimondo (2008) [19]	Prospective monocentric	3	13	0	15	0	ROC based	Cortisol at 15′: 44 nmol/L (1.6 μg/dL)	RIA	94	93	210	0.5 mg every 6 h, 8 doses	Ovine	100 µg
Valassi (2009) [20]	Retrospective, monocentric	1	59	0	41^$^		Predefined	Cortisol at 15′: 38 nmol/L (1.4 μg/dL)	C.L. assay	86	85	35	0.5 mg every 6 h, 8 doses	Ovine	1 µg/kg
Alwani (2014) [21]	Prospective, monocentric	0	35	0	19	0	ROC based	Cortisol at 15′: 87 nmol/L (3.2 μg/dL)	C.L. assay	94	100	523	0.5 mg every 6 h, 8 doses	Human	1 µg/kg

*CS* Cushing’s syndrome, *ACS* Adrenal Cushing’s syndrome, *CD* Cushing’s Disease, *EAS* Ectopic ACTH secreting tumor, *NNH* non-neoplastic hypercortisolism, *pCS* pseudo-Cushing related condition, *rc* related condition, *ex* excluded, *ROC* Receiving Operator Characteristic, *RIA* radio-immunometric assay, *C.L.* chemi-luminescent, *Se* Sensitivity, *Sp* Specificity, *DOR* Diagnostic odds ratio, *Dex* Dexamethasone, *iv* intravenous

*Most of the patients received human CRH, while 6 out of 31 (19.3%) received ovine CRH

^#^Derived from Fig. 5 of the original publication

^¥^Paediatric subjects

^§^For a maximum of 500 µg

^$^Proportion not specified

**Table 2:** Studies included in the metanalysis for Desmopressin test

Study	Design, setting	CS			NNH/pCS		Cut-off			Se (%)	Sp (%)	DOR	iv Desmopressin
		ACS	CD	EAS	pCS rc	CS ex	Type	Value	Assay				
Moro (2000) [22]	Prospective, monocentric	0	20	0	30	0	ROC based	Peak Δ-ACTH ≥ 6 pmol/L	RIA	90	97	261	10 µg
Pecori-Giraldi (2007) [17]	Prospective, multicentric	0	27	0	21	0	Predefined	Δ-ACTH ≥ 6 pmol/L 0’–30′	RIA	81	90	42	10 µg
Tirabassi (2010) [23]	Retrospective, monocentric	0	23	0	28	0	Predefined	Basal serum cortisol > 331 nmol/L (12 μg/dL) and Δ-ACTH > 4 pmol/L (18 pg/mL)	C.L. assay	87	93	87	10 µg
Rollin (2015) [24]	Prospective, monocentric	0	68	0	56	0	ROC based	Peak ACTH of 15.8 pmol/L	C.L. assay	91	95	183	10 µg

*CS* Cushing’s syndrome, *ACS* Adrenal Cushing’s syndrome, *CD* Cushing’s Disease, *EAS* Ectopic ACTH secreting tumor, *NNH* non-neoplastic hypercortisolism, *pCS* pseudo-Cushing, *rc* related condition, *ex* excluded, *ROC* Receiving Operator Characteristic, *RIA* radio-immunometric assay, *C.L.* chemi-luminescent, *Se* Sensitivity, *Sp* Specificity, *DOR* Diagnostic odds ratio, *iv* intravenous

**Table 3:** Studies included in the metanalysis for CRH (corticotropin releasing hormone) test

Study	Design, setting	CS			NNH/pCS		Cut-off			Se (%)	Sp (%)	DOR	iv CRH	
		ACS	CD	EAS	pCS rc	CS ex	Type	Value	Assay				Type	Dosage
Yanovski (1993) [7]	Prospective, monocentric	2	35	2	19	0	ROC-based	Sum of post-CRH cortisol levels > 3450 nmol/L (125 μg/dL)	RIA	64	100	67	Ovine	1 µg/kg
Tirabassi (2011) [25]	Retrospective, monocentric	0	30	0	18	12	Predefined	Basal serum cortisol > 331 nmol/L (12 μg/dL) and ACTH peak > 12 pmol/L (54 pg/mL)	C.L. assay and RIA	97	100	1120	Human	100 µg
Ceccato (2020) [26]	Retrospective, monocentric	0	86	0	9	22	ROC-based	Peak cortisol > 685.5 nmol/L (20.6 µg/dL) and peak ACTH > 15.4 pmol/L (70 pg/mL)	C.L. assay	84	65	9	Human	100 µg

*CS* Cushing’s syndrome, *ACS* Adrenal Cushing’s syndrome, *CD* Cushing’s Disease, *EAS* Ectopic ACTH secreting tumor, *NNH* non-neoplastic hypercortisolism, *pCS* pseudo-Cushing, *rc* related condition, *ex* excluded, *ROC* Receiving Operator Characteristic, *RIA* radio-immunometric assay, *C.L.* chemi-luminescent, *Se* Sensitivity, *Sp* Specificity, *iv* intravenous, *DOR* Diagnostic odds ratio

### Study characteristics

For Dex-CRH we identified 9 eligible studies (2 multicentric and 7 monocentric); five presented a prospective design and the remaining four were retrospective series (Table 1). Overall, we considered 449 patients, 243 with CS (13 ACS, 228 CD, 2 EAS) and 206 without CS (132 with pCS-related conditions, 33 CS-excluded, 41 unknown). In the work from Valassi et al. [20] it was not possible to discriminate the correct proportion between patients with actual NNH/pCS (i.e., presenting a pCS related condition) and CS-excluded subjects in the NNH/pCS group. All studies except one used ROC-based criteria. Most studies considered cortisol-based cut-offs, while Gatta et al. [15] found equally performing cortisol and ACTH-based thresholds and Erickson et al. [16] preferred the ACTH-based cut-off. Both chemi-luminescent and radio-immunometric assays were utilized. Dexamethasone schedule and dosage were mainly based on the original work from Yanovski et al. [7] providing 8 oral administrations of Dexamethasone 0.5 mg over 2 days; Martin et al. [14] included an additional dose and Batista et al. [18] adjusted the dosage for their paediatric population. CRH type and dosage varied across the studies.

For desmopressin test we considered four studies, three with a prospective design; one of them presented a multicentric setting (Table 2). Overall, 138 CS patients and 135 NNH/pCS patients were included; no ACS, EAS or CS-excluded patients were reported in these series. For half of the included studies a ROC-based cut-off was considered, for the remaining two a predefined cut-off was preferred. All studies provided ACTH-based cut-offs, except for Tirabassi’s one [23] that presented a combined threshold with both ACTH and cortisol to optimize the performance. The authors applied both chemi-luminescent and radio-immunometric assays. All studies used iv desmopressin at the dosage of 10 µg.

For CRH test only three studies met the inclusion criteria; they were based on monocentric series, two presenting a prospective design and the third a retrospective one (Table 3). Overall, 155 CS patients (2 ACS, 151 CD, 2 EAS) and 80 NNH/pCS patients (46 with pCS-related conditions, 34 CS-excluded) were considered for analysis. In Ceccato’s work [26] the distinction between clinically characterized NNH/pCS and CS-excluded patients was not available, but it was possible to derive it from the reported original database. Only one study used a predefined cut-off, the others applied ROC-based thresholds. Both chemi-luminescent and radio-immunometric assays were used. While Yanovski and coll [7] used 1 µg/kg iv of ovine CRH, both the other studies preferred 100 µg of human CRH iv as the stimulus.

No study reporting Dex-Desmopressin test met the inclusion criteria.

### Risk of bias

**Fig. 3:**
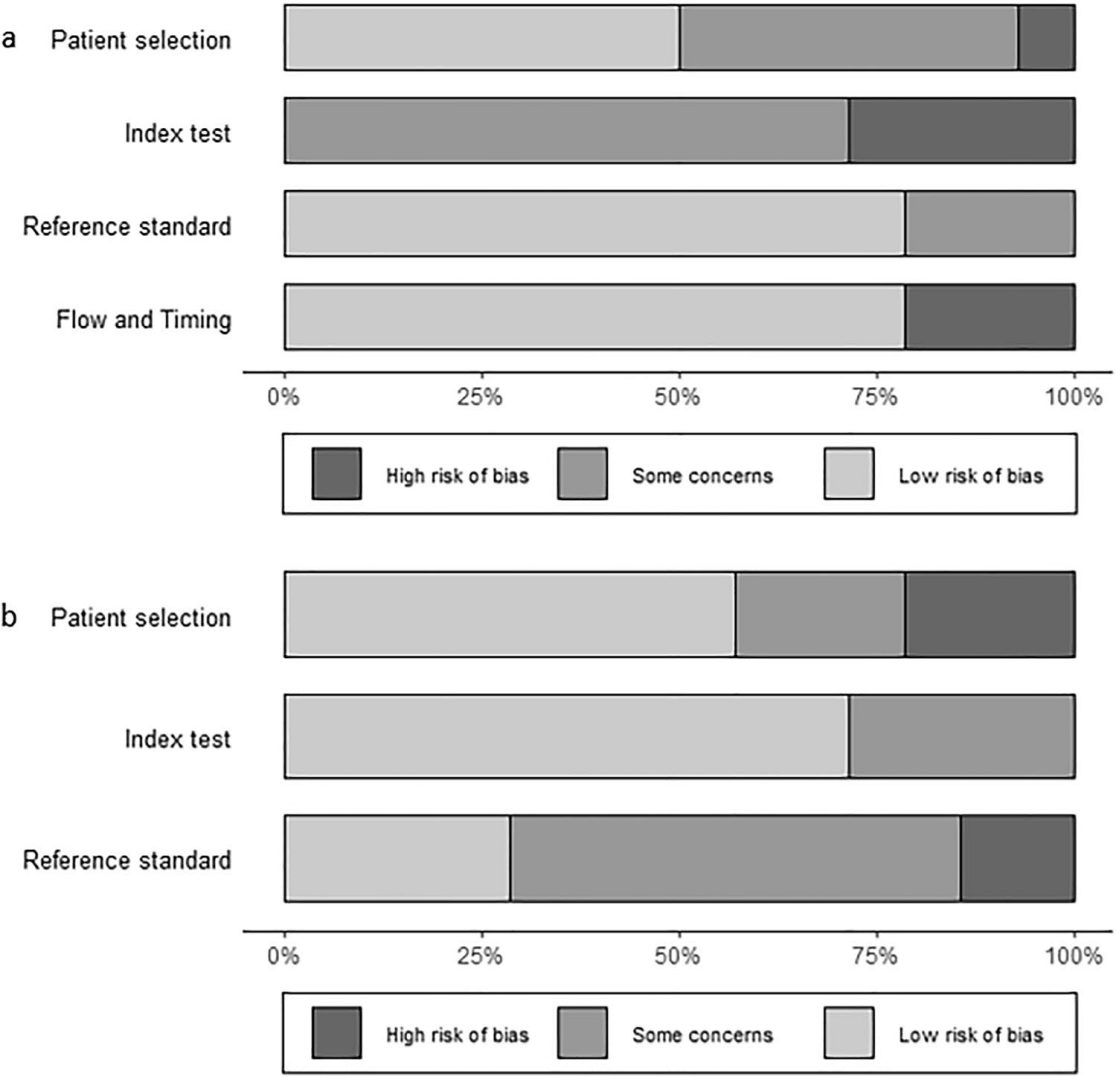
Application of QUADAS-2 tool in three domains (patient selection, index test, reference standard) for both risk of bias (**a**) and applicability concern (**b**) assessments. The risk of bias (**a**) was also evaluated in a fourth domain regarding flow and timing throughout the study

In the evaluation of the risk of bias was performed using QUADAS-2 tool (Fig. 3), as expected, most works presented sources of bias and concerns about applicability across various domains. The most affected domain was the risk of bias for index test. It was not possible to evaluate the ratio of CS-excluded patients in the NNH/pCS group in the series presented by Valassi et al. [20], thus the applicability concern for patient selection was prudently deemed high. In Batista’s paper [18], per protocol assessment was revised due to additional considerations. Although patient selection presented a per protocol low risk of bias, some concerns were addressed due to the evaluation of a paediatric population. As paediatric response to CRH stimulation was reported to be more pronounced [27], some concerns also rose regarding applicability of the index test.

### Metanalysis

Dex-CRH test provided sensitivity of 97% (95 CI% [88%; 99%]), specificity of 92% (95% CI [84%; 96%]) and diagnostic odds ratio of 138.83 (95% CI [49.38; 390.32]). Small heterogeneity was found for each parameter (i.e., I^2^ < 50%). Egger’s regression did not address significant publication bias (intercept = 1.85, t = 1.24, p = 0.304).

Desmopressin test provided sensitivity of 88% (95% CI [77%; 95%]), specificity of 94% (95% CI [83%; 98%]) and diagnostic odds ratio of 110.44 (95% CI [32.13; 379.63]). No heterogeneity was found across the considered indexes (I^2^ = 0%). Egger’s regression did not address significant publication bias (intercept = 2.45, t = − 0.97, p = 0.434).

CRH test provided sensitivity of 84% (95% CI [30%; 99%]), specificity of 99% (95% CI [0%; 100%]) and diagnostic odds ratio of 64.77 (95% CI [0.15; 27,174.73]). High heterogeneity emerged for all parameters except specificity (I^2^ = 0%). Egger’s regression did not address significant publication bias (intercept = 1.93, t = − 0.43, p = 0.723).

**Fig. 4:**
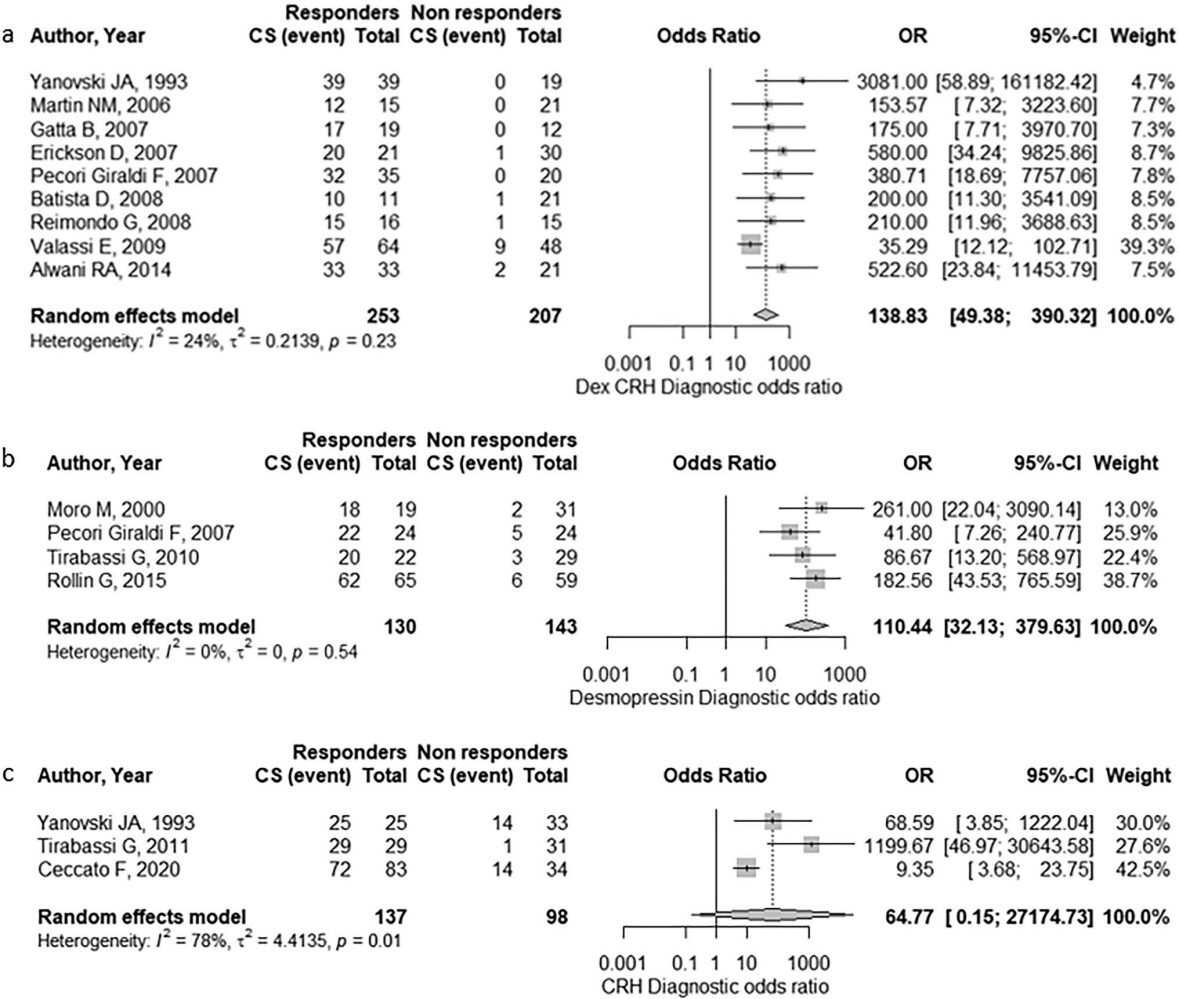
Pooled effect for diagnostic odds ratio of Dex-CRH test (**a**), Desmopressin test (**b**) and CRH test (**c**). *CS* Cushing’s syndrome, *Dex* dexamethasone, *CRH* corticotropin realising hormone, *OR* odds ratio, *CI* confidence interval

Forest plots for diagnostic odds ratio are showed in Fig. 4. Forest plots for sensitivity and specificity, as well as funnel plot analysis for each test, are reported in the Supplementary Data [13].

### Metaregression

**Fig. 5:**
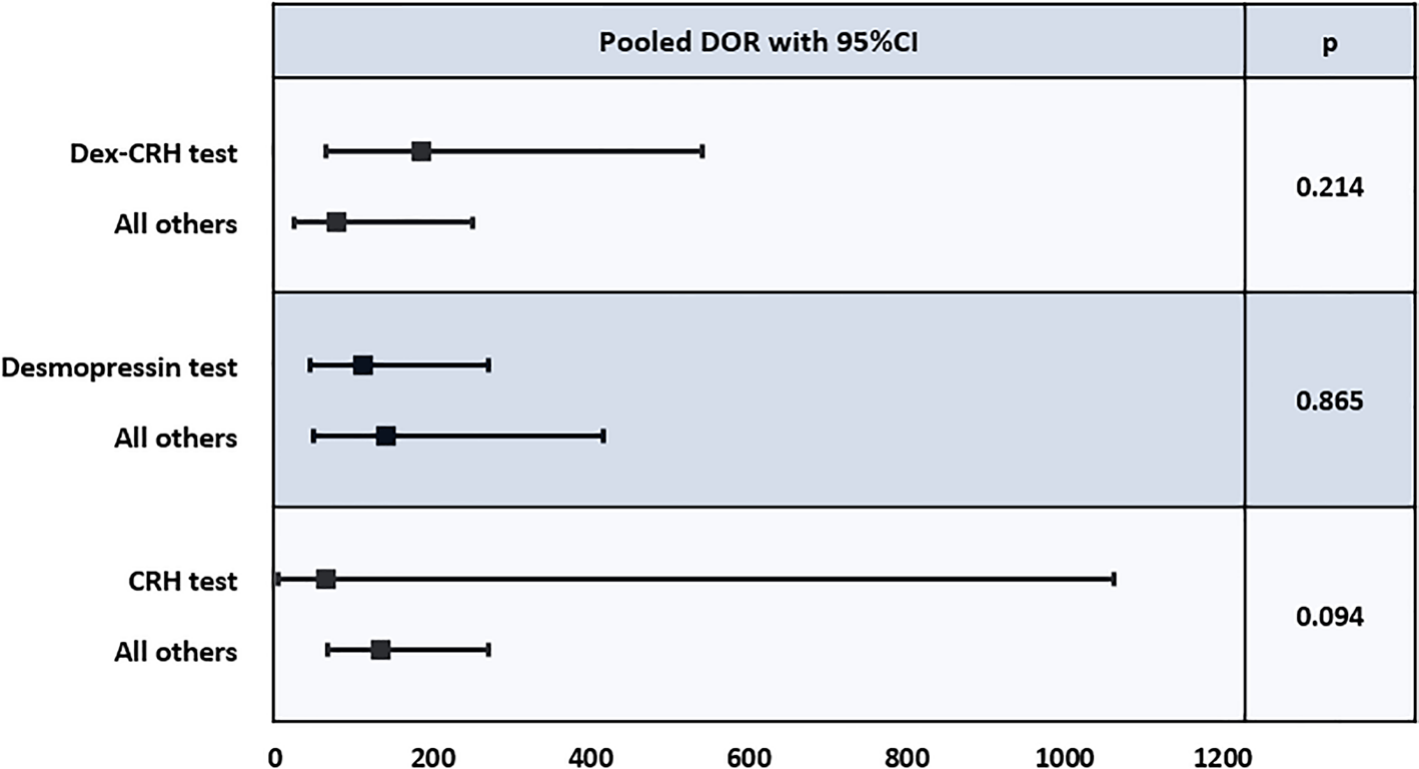
Metaregression analysis for diagnostic odds ratio. For each comparison (index test chosen versus all other index tests) pooled diagnostic odds ratio, confidence intervals and p-value are reported. *DOR* diagnostic odds ratio, *95% CI* 95% confidence interval, *Dex* dexamethasone, *CRH* corticotropin releasing hormone

The metaregression analysis did not indicate a significantly superior test in terms of diagnostic odds ratio (Fig. 5), sensitivity or specificity (data not shown). The metaregression analysis was also performed on Dex-CRH test, comparing data based on the different stimulus applied (i.e., human versus ovine CRH), without significative differences (data not shown). Gatta’s work [15] was included in the human CRH group for this analysis.

## Discussion

The clinical and hormonal overlap between NNH/pCS and CS patients is a well-known challenge, especially when first line tests for hypercortisolism show mild alterations. The recent consensus on CD suggested that UFC is almost always within threefold the upper limit of normal in case of functional hypercortisolism [4]. An adequate clinical follow-up and the repetition of first line tests over time, especially when a pCS related condition can be treated, are of the outmost importance to clarify the picture. Some cases may be sorted out in few months, but longer follow-up is often required. Therefore, various second line tests that had been proposed to early differentiate these conditions. In order to enhance discrimination between functional and neoplastic hypercortisolism, these tests are based on the stimulation of the HPA axis, with or without prior Dex suppression. However, a consensus on the gold standard among them is still missing.

Dex-CRH test was firstly investigated in this setting from Yanovski’s original work [7], based on the hypothesis that only CS patients could maintain cortisol response to CRH stimulation after Dex suppression. The reported results showed perfect discrimination between NNH/pCS and CS patients (i.e., sensitivity 100%, specificity 100%). No following series reproduced these results, independently of the cut-off adopted. Nevertheless, this test performed well overall, with both sensitivity and specificity above 90%. In particular, the sensitivity resulted high, although the finding may be partly due to the selection criteria of our analysis (detailed in “Materials and methods”). Although scarce heterogeneity was addressed by the I^2^, the index test application was highly variable, with differences in dexamethasone dosage and schedule and in CRH type and dosage (Table 1). Moreover, the Nieman’s editorial [28] previously addressed various confounders about this topic, such as high interpersonal variability in dexamethasone metabolism, the not always reliable compliance to dexamethasone schedule in the outpatient setting and the low accuracy of cortisol determination at low levels (especially for assay methods based on antibodies). In Valassi’s work [20], patients assuming medications presumed to interfere with dexamethasone metabolism presented a reduction in the specificity of post-CRH cortisol value, especially in case of multiple interfering treatments. Unfortunately, dexamethasone levels we available only for a part of the patients, but an abnormal dexamethasone level could explain some equivocal responses after Dex suppression. Among other articles considered in our analysis, only Reimondo et al. [19] deployed this tool, with post-suppression dexamethasone levels presenting similar range to that of healthy volunteers receiving the same test as controls. Systematic assessment of dexamethasone levels after suppression may optimize test performance, as previously shown for low dose DST in the diagnosis of CS [29]. CRH is an expensive compound with limited availability around the globe, especially in countries with limited resources. Moreover, a further accessibility issue emerged in December 2022, with the announcement that Ferring Pharmaceuticals would stop the production of CRH due to technical problems. It has been estimated that the shortage will extend for 36 months [30], requiring physicians to reroute some well-established algorithms for HPA axis diseases. At our centre, despite higher costs, we retrieved some vials of a human CRH produced in Japan to ensure a complete work-up only for extremely selected patients, and after a multidisciplinary discussion. Despite the above-mentioned pitfalls, Dex-CRH seems to be a solid diagnostic tool in this challenging differential diagnosis.

Desmopressin test is based on the evidence of vasopressin receptor 2 (VR-2) expression in ACTH-secreting adenomas. Thus, the synthetic analogue desmopressin should elicit ACTH response only in CD patients, as the normal pituitary mainly expresses VR-3 with scarce affinity for this stimulus [8]. Overall I^2^ analysis did not address significant heterogeneity. This test also showed satisfying performance, with specificity well above 90%. It should be mentioned that data on the desmopressin, compared to Dex-CRH test, are more focused on our review question (i.e., only CD and NNH/pCS patients are included for its analysis) and index test protocol was standardized across the studies (i.e., 10 µg of iv desmopressin). This test is less complex than Dex-CRH and may be more suitable for clinical practice. Moreover, desmopressin is less expensive and more widely available than CRH.

CRH test is widely used in discriminating the source of ACTH excess in ACTH-dependent CS [31]. In our setting, it showed excellent specificity but seemed overall lacking in sensitivity. 95% CIs were extremely large for this test, probably reflecting both the limited number of studies included and the heterogeneity of the results (also addressed by the I^2^ index). Moreover, recent studies preferred a different stimulus compared to the older Yanovski’s work [7] (Table 3). Indeed, previous dexamethasone suppression (i.e., Dex-CRH test) may prevent effective corticotroph response in NNH/pCS patients, enhancing their discrimination from neoplastic hypercortisolism.

Although metaregression analysis based on diagnostic odds ratio failed to provide a gold standard, CRH test seemed to lack in performance compared to the other tests. Dex-CRH seemed to perform slightly better than Desmopressin test, but further considerations cannot be carried out due to large 95% CIs.

In Pecori-Giraldi’s work [17] both the last-mentioned tests did not provide perfect discrimination between NNH/pCS and CS. In line with the result of our metanalysis, Dex-CRH provided better sensitivity, while desmopressin test presented better specificity. Desmopressin test in combination with results from the DST (providing high sensitivity in their series) proved to be superior to Dex-CRH alone. This finding hints to another possible approach, that of a Dex-Desmopressin test. Although the work from Araya et al. [9] did not meet the inclusion criteria for this meta-analysis, Dex-Desmopressin showed good performance in their cohort and may be a promising tool for further investigations.

Unfortunately, our study presents many limitations, reducing the strength of its conclusions. The main flaws of our analysis are the limited number of studies available, especially those including CRH, as well as the heterogeneity in both study design (i.e., prospective and retrospective series) and populations recruited (i.e., inclusion of CS-excluded patients, heterogeneity in cortisol-related conditions, inclusion of non-pituitary CS). These factors led to large confidence intervals, and reduced statistical power. As reported in the Cochrane handbook for Handbook for Systematic Reviews of Interventions [32], the width of a confidence interval for a meta-analysis depends both on the precision of the individual study estimates and on the number of studies combined. The precision of an individual study is strongly influenced by its sample size, with larger studies tending to give more precise estimates (i.e., narrower CIs) than smaller studies. Hence, our analysis is negatively affected from both the low number of studies included and their individually small population. One of the possible reasons behind the lack of studies on second line tests on NNH/pCS may be the relatively novelty of this topic, starting from the work of Yanovski in the early 1990s [7]. Different laboratory assays, CRH formulation and dosage, and dexamethasone schedule were also sources of heterogeneity, with the QUADAS-2 tool presenting an especially high risk of bias regarding the index test domain. The inclusion criteria focused on ROC-based cut-offs inevitably reinforced the issue.

Despite the above-mentioned flaws, our analysis suggests, in line with the most recent consensus [4], that both Dex-CRH and Desmopressin test can be valid tools in helping the discrimination between NNH/pCS and CS. The first probably achieves a slightly better performance and the latter seems more suitable for clinical practice. Data regarding CRH are surely lacking and based on existing evidence we cannot recommend it alone in this specific setting, although its combination with desmopressin test showed promising results and could be further studied [25]. To date, although a skilled application of the dynamic tests can be useful, the gold standard for correctly identifying NNH/pCS patients should still be the reversal of hypercortisolism by treating identifiable causes or the lack of clinical and biochemical progression during an adequate follow-up [2]. Referral to an endocrinology centre with specific expertise is advisable, possibly with a re-assessment of the initial diagnosis of hypercortisolism. Indeed, mass spectrometry-based assays may help to avoid unnecessary further testing on patients presenting falsely positive first level hormonal analyses [33, 34].

It is important to correctly differentiate NNH/pCS and CS, as the latter is burdened by increased morbidities and mortality [35]. Nevertheless, the consequences of a functional hypercortisolism are not clear to date: an exposure to mild biochemical hypercortisolism may negatively impact on the patient’s health, as reported for the mild autonomous cortisol secretion in adrenal adenomas [36, 37].

Further studies are needed on this topic, possibly focusing on mild CD and clinically well-characterized NNH/pCS patients. It may be useful to study each pCS related condition individually, in order to obtain insights for a tailored testing approach.

## Data Availability

All data generated or analysed during this study are included in this published article or in its supplementary data ([13] https://researchdata.cab.unipd.it/id/eprint/827).
